# Exploring major signaling cascades in melanomagenesis: a rationale route for targetted skin cancer therapy

**DOI:** 10.1042/BSR20180511

**Published:** 2018-10-02

**Authors:** Paola M. Dantonio, Marianne O. Klein, Maria Renata V.B. Freire, Camila N. Araujo, Ana Carolina Chiacetti, Ricardo G. Correa

**Affiliations:** 1 Department of Cell and Developmental Biology, Institute of Biomedical Sciences, University of Sao Paulo, Sao Paulo, Brazil; 2 Department of Anatomy, Institute of Biomedical Sciences, University of Sao Paulo, Sao Paulo, Brazil; 3 Radiopharmacy Center, Nuclear and Energy Research Institute, University of Sao Paulo, Sao Paulo, Brazil; 4 Department of Microbiology, Institute of Biomedical Sciences, University of Sao Paulo, Sao Paulo, Brazil; 5 Department of Immunology, Institute of Biomedical Sciences, University of Sao Paulo, Sao Paulo, Brazil; 6 Center for Translational Neuroscience, Sanford Burnham Prebys Medical Discovery Institute, La Jolla, CA, U.S.A.

**Keywords:** intracellular signaling, melanoma, melanomagenesis, skin cancer

## Abstract

Although most melanoma cases may be treated by surgical intervention upon early diagnosis, a significant portion of patients can still be refractory, presenting low survival rates within 5 years after the discovery of the illness. As a hallmark, melanomas are highly prone to evolve into metastatic sites. Moreover, melanoma tumors are highly resistant to most available drug therapies and their incidence have increased over the years, therefore leading to public health concerns about the development of novel therapies. Therefore, researches are getting deeper in unveiling the mechanisms by which melanoma initiation can be triggered and sustained. In this context, important progress has been achieved regarding the roles and the impact of cellular signaling pathways in melanoma. This knowledge has provided tools for the development of therapies based on the intervention of signal(s) promoted by these cascades. In this review, we summarize the importance of major signaling pathways (mitogen-activated protein kinase (MAPK), phosphoinositide 3-kinase (PI3K)-Akt, Wnt, nuclear factor κ-light-chain-enhancer of activated B cell (NF-κB), Janus kinase (JAK)-signal transducer and activator of transcription (STAT), transforming growth factor β (TGF-β) and Notch) in skin homeostasis and melanoma progression. Available and developing melanoma therapies interfering with these signaling cascades are further discussed.

## Introduction

Cutaneous (skin) melanoma is one of the most aggressive and treatment-resistant human cancers [1]. Indeed, skin melanoma is characterized by a high tendency to metastasize since it is located in the basal layer of the epidermis, which is constituted by several blood and lymphatic vessels [2,3]. In this sense, although melanoma represents less than 5% of skin cancers, it has a high social impact which accounts for 80% of cancer-related deaths, being highly lethal [4].

Melanoma is considered a form of malignant tumor (cancer) that affects the melanin-producing cells, also known as melanocytes. In humans, melanocytes can be detected in the epidermis around the 50th day of intrauterine life [5]. Embryonically, these cells migrate from the neural crest to the basal layer of epidermis along the dorsolateral pathway [6]. As melanoma precursors, it has been suggested that the melanocyte developmental program is frequently utilized by the tumor cells to promote its own progression [7].

Melanocytes are found across the whole body, including the skin, iris (eye), cochlea (ear), mucosa, and others [8,9]. By distributing pigment from melanosomes (melanin-containing organelles) to keratinocytes present in the skin, melanocytes has a protective role against UV radiation (UVR) [2]. The entire spectrum of UVR has been implicated in the pathogenesis of melanoma, since it can cause mutations in skin cells by direct interaction with DNA or indirectly through the generation of reactive oxygen species (ROS). Therefore, UVR has been recognized by the World Health Organization as carcinogenic to humans [10]. In fact, extensive sunlight exposure (UVA and UVB radiation) is the major risk factor for cutaneous melanoma, and leads to a genetic signature that is characteristic of this disease [9,10]. Artificial tanning, for instance, is a relatively recent social phenomenon for certain societies involving high UV light exposure, which certainly adds up genotoxic effects to skin cells and potentially leads to DNA damage [11,12].

Phenotypically, white populations with fair skin (mainly those with red hair and freckles) have higher risk of developing melanoma [13]. However, besides environmental and phenotypic predisposition, melanoma also arises from genetic susceptibility. Family history and the presence of nevi are some of the most important risk factors for melanoma. Familial melanoma is responsible for 8–12% of melanoma cases [14], being cyclin-dependent kinase inhibitor 2A (*CDKN2A*) and *CDK4* the major susceptibility genes involved in this context [15]. Germline mutations in *CDKN2A* are responsible for the loss of two tumor suppressor proteins, p16^INK4a^ and p14^ARF^, both encoded by the *CDKN2A* gene through alternative splicing [16,17], while germline mutations in the *CDK4* oncogene render a constitutively active complex between CDK4 and cyclin D1, which in turn promotes abnormal proliferation [18].

Melanocytic nevus is a benign accumulation of melanocytes that can be considered the first lesion in melanoma progression, and can develop to melanocytic hyperplasia and ultimately dysplasia [19,20]. Dysplastic tumors can further progress through non-invasive and invasive lesions until metastatic melanoma is established. This multistep tumorigenic process results from the accumulation of genetic alterations that comprise genomic instability, activation of oncogenes such as *BRAF* and *RAS*, as well as inactivation of tumor suppressor genes (TSGs) [21]. For instance, allelic deletions at chromosomes 1p, 6q, 9p 11q, 17q, and others can be found in melanoma [22]; some of these regions harbor melanoma susceptibility genes and other TSGs [23,24], rendering their loss an oncogenic event. Oncogenes may also be amplified in melanoma, as is the case of *CCDN1, KIT* and telomerase reverse transcriptase (*TERT*) [25].

Both the incidence and the annual mortality due to melanoma have increased dramatically during the last few decades [26]. It is estimated that the number of cases worldwide has doubled in the past 20 years, and it has been reported that the incidence of melanoma has increased faster than any other type of cancer (~3.1% per year) [27,28]. Cutaneous melanoma is the fifth most frequent cancer in men and sixth in women, with an estimate of 55000 and 36000 new cases in 2018 in the United States, respectively. Thus, it is estimated that the probability of a man developing cancer during his lifetime is 1.72% and for a woman is 1.22% [29].

Australia and New Zealand have the highest incidence rates of cutaneous melanoma, reaching ~60 cases per 100000 inhabitants/year. However, these rates vary vastly amongst different populations: the United States has a rate of ~30 cases per 100000 inhabitants per year while in Europe it is ~20 cases per 100000 per year [8]. In Latin America, there is a lack of data regarding the incidence and prevalence of melanoma [30]. Considering that Latin Americans are frequently exposed to high amounts of UVR due to high altitude and tropical climate, it would be important to better distinguish the impact of this disease in these populations. Most studies in countries such as Argentina, Brazil, Mexico, and Colombia have been conducted in hospitalized patients and, therefore, do not reflect the incidence and prevalence of the disease in their population as a whole [30,31]. As an example, the National Institute of Cancer in Brazil (INCA) estimates 6260 new cases of melanoma in Brazil in 2018 [30,32].

Clinically, four main subtypes of melanoma have been described: nodular, acral lentiginous melanoma (ALM), lentigo maligna (LM), and superficial spreading melanoma (SSM) [27]. Nodular melanoma consists of raised nodules without a significant flat portion; ALM tends to be found on the palms of the hands, the soles of the feet, and in the nail bed; LM is generally flat in appearance and occurs on sun-exposed regions in the elderly, being related to lifetime chronic sun exposure [27]. SSM is the most common form of melanoma, and it is frequently linked to episodes of severe sunburn (especially at an early age). Thus, it mostly occurs on areas of the body with periodic sun exposure, such as the trunk and proximal extremities [2,27].

Clinical staging of melanoma progresses from a focal (*in situ*) growth, followed by increasing thickness and vertical invasion to regional lymph-node spread and, ultimately, resulting into distal metastasis [2]. Most melanoma cases are localized at the time of diagnosis and, at early stages, may be cured by surgical resection [33,34]. However, metastatic melanoma is extremely hard to treat, being unresponsive to most of the existing therapies and associated with a poor prognosis, with a median survival rate of 6–12 months and 5-year survival rate of less than 5% [8,27,33]. A variety of biological mechanisms can impair the therapy for metastatic melanoma and also lead to drug resistance, such as over-activated pro-survival signaling pathways, triggering of alternative compensatory pathways, increased expression of therapeutic target(s), molecular heterogeneity, and up-regulation of drug transporters [35].

Analysis of The Cancer Genome Atlas (TCGA) and other genome-wide data [36,37] made possible to identify the most frequent mutations in melanoma and other types of cancers. BRAF^V600E^ is the most prevalent mutation in melanoma, being detected in 52% of cases, followed by mutations in the *RAS* family and neurofibromin 1 (*NF1*) in approximately 30 and 14% of cases, respectively [36]. These driver mutations are almost always mutually exclusive, making it possible to classify melanoma cases in distinct genomic subtypes: *BRAF, RAS, NF1*, and Triple-WT, the latter being defined by the absence of *BRAF, RAS*, and *NF1* mutations. Other common alterations in melanoma include mutations in *TP53, CDKN2A*, and phosphatase and tensin homolog (*PTEN*) TSGs and in *TERT* promoter [36].

Recent discoveries in cell signaling mechanisms have provided better understanding of the biology that underlies melanoma progression, and these advances have been exploited to provide targetted drugs and new therapeutic approaches [27]. Among the genetic events that underlie melanoma development, a variety of mutations in cell signaling pathways components has been characterized. Many of these alterations impair receptor functions at the plasma membrane and the emanating signaling cascades [2]. In the present review, we summarize the roles and altered functions of major signaling pathways during melanoma development, also providing a detailed description of the available and under-development treatments that may impact these cascades.

## Mitogen-activated protein kinase pathway

The mitogen-activated protein kinase (MAPK) cascade is an evolutionarily conserved signal transduction pathway, involved in a variety of physiological programs, such as cell proliferation, differentiation, development, migration, apoptosis, and transformation [38,39]. This pathway can be activated by a broad range of extracellular signals – growth factors, mitogens, cytokines, and others – that will lead to temporal and tissue-dependent biological effects in the organism. Fourteen MAPKs have been identified in mammals, and these kinases are typically divided in three main subfamilies: the extracellular signal-related kinases (ERKs), Jun N-terminal kinases (JNKs), and p38 kinases [40]. Each of these MAPKs is activated upon phosphorylation by an MAPK kinase (MAPKK or MAP2K), which in turn is activated by an MAPKK kinase (MAPKKK or MAP3K). There are at least seven MAPKKs (MAPK/ERK kinase (MEK) 1–7 (MEK1–7)) and several MAPKKKs described so far [41]. MAP3Ks include proteins from numerous families, such as the Raf isoforms, 1001-amino acid protein (Tao) and Mos proteins, and others [41]. These kinases ultimately drive the activation of ERK, JNK, or p38 MAPKs, which will prompt distinct cellular responses by further activating specific substrates and transcription factors.

The ERK pathway is the best characterized MAPK pathway in mammalian cells and, coincidentally, it has an important impact on melanoma development and progression. In this MAPK axis, the MAP3K role is played by the Raf family of serine/threonine kinases, which is characterized by an Ras/GTP-binding domain [42]. Ras proteins (H-Ras, N-Ras, and K-Ras) are small GTPases located in the plasma membrane that act as activators in several pathways besides MAPK. Ras oscillates between its ‘off’ GDP-bound and ‘on’ GTP-bound states [43]. This oscillation is facilitated by guanine nucleotide exchange factors (GEFs) and GTPase-activating proteins (GAPs), which mediates GTP binding and GTP hydrolysis, respectively.

**Figure 1:**
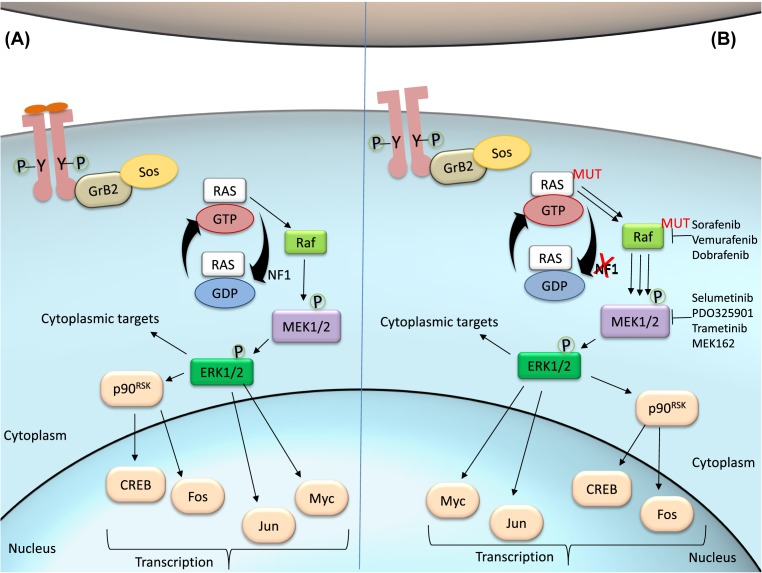
The MAPK-ERK pathway (**A**) In regular conditions, ligands such as growth factors or mitogens bind to the RTK, which is activated by autophosphorylation. Phosphotyrosine residues recruit adaptor protein Grb2 and Sos, promoting Ras:GTP association. Activated by GAPs such as NF1, Ras hydrolyzes GTP and activates Raf, the first effector kinase in the MAPK pathway. Raf then phosphorylates MEK, which in turn phosphorylates ERK. p-ERK activates cytoplasmic and nuclear substrates. (**B**) In melanoma cells, mutations in Ras may impair its capacity to hydrolyze GTP, therefore remaining activated. Loss of function mutations on NF1 are involved in the same mechanism, promoting sustained Ras:GTP association. Mutations in B-Raf kinase are the most frequent in melanoma, causing continued phosphorylation of MEK and downstream targets. The pathway can be targetted at any level by specific inhibitors.

Upon ligand binding, receptor tyrosine kinase (RTK) undergoes dimerization and cross-phosphorylation at one or more tyrosine residues [44], which will then serve as an assembly and activation site for downstream intracellular proteins. In the MAPK pathway, phosphotyrosine residues at the cytoplasmic domain of the receptors serve as docking sites for the adaptor protein growth factor receptor-bound protein 2 (Grb2), which further recruits the GEF son of Sevenless (Sos) and triggers the GTP loading of Ras (Figure 1A). Ras/GTP recruits Raf kinases to the plasma membrane for further activation: when Ras is stimulated by GAPs, it binds to Raf and GTP is hydrolyzed into GDP, resulting in the activation of Raf [45]. A-Raf, B-Raf, and C-Raf are upstream effectors of the MAPK pathway and phosphorylate the MAPKKs MEK1/2. The Raf isoforms possess distinct capacity to activate MEKs, being B-Raf the strongest activator. Phosphorylated and active MEKs are then capable of phosphorylating ERK1/2, the final effectors of the MAPK/ERK pathway. ERK activation results in the phosphorylation of a plethora of substrates, both in the cytoplasm and in the nucleus. ERK localization will determine target substrates. In the cytosol, ERK can phosphorylate cytoskeletal proteins and modulate cell movement, trafficking, and adhesion [46]. In the nucleus, ERK can activate a variety of transcription factors such as p90^RSK^, c-Fos, c-Myc, and c-Jun [47,48].

### Activity of MAPK pathway in melanoma

Normal epidermal cells are constantly replenished to sustain the cutaneous barrier, and a few studies have shown that MAPK pathway plays a significant role at this steady proliferative condition [49,50]. In melanocytes, MAPK is also involved in the balance between proliferation and differentiation [51]. For instance, the microphthalmia-associated transcription factor (MITF) is a target of ERK and regulates melanocyte functions such as melanin production, survival, and cell fate [52], which are dependent on the pattern of ERK activation. Transient activation of ERK can induce melanocyte differentiation [53,54], while sustained activation promotes melanocyte proliferation [55]. Therefore, dysregulated MAPK signaling and sustained ERK activation are intimately related to melanoma progression.

During cancer progression, the MAPK pathway may be compromised at distinct stages (Figure 1B). Alterations on the pathway, including mutations of certain effector molecules, may eventually lead to cascade hyperactivity and subsequent biological effects such as cell proliferation, survival, invasion, metastasis, and angiogenesis [56,57]. The *BRAF* gene is frequently mutated in several cancers, and *BRAF^V600E^* is the most common mutation in skin melanoma [36]. Mutated *BRAF^V600E^* leads to an elevated kinase activity of B-Raf and sustained activation of downstream targets, in addition to unresponsive negative feedback mechanisms [58]. The Q61 mutant Ras, the most predominant in melanoma [59], leads to an important decrease in its intrinsic hydrolytic activity [60] and therefore a sustained active state of Ras. Mutations in other molecules may also lead to Ras overstimulation, such as loss-of-function mutations in *NF1* [61]. Neurofibromin, the protein encoded by the *NF1* gene, acts as a GAP and promotes the hydrolysis of Ras/GTP into Ras/GDP, with consequent Ras inactivation [62]. In melanoma, most cases with altered *NF1* comprise loss-of-function mutation [36], in which neurofibromin loses its capacity to inactivate Ras and promotes sustained stimulation of Raf and its downstream targets, leading to overstimulation of the MAPK pathway and consequent cell growth, proliferation, and survival [63].

In addition to MITF, the AP-1 family of transcription factors (which includes c-Jun and c-Fos) is also a direct target of MAPK signaling, and it is highly associated with melanoma progression, proliferation, migration, apoptosis, and cell survival [64,65]. Indeed, c-Jun is considered the most important AP-1 factor in melanoma and it can be activated by both JNK and ERK MAPKs to promote cell proliferation [57]. Several c-Jun target genes have been implicated in tumorigenesis and are typically deregulated in many types of cancer, including melanoma [66]. FOSL1, another AP-1 factor, has also been related to melanoma progression by enhancing the expression of pro-tumorigenic genes, cell migration, and proliferation [67].

According to the TCGA data analysis, *TERT* promoter mutations frequently occur in melanoma, mainly in the *BRAF* (75% of cases), *RAS* (72% of cases), and *NF1* (83% of cases) subtypes [36], suggesting a link between MAPK activation and *TERT* expression. Indeed, constitutively active MAPK pathway promotes phosphorylation and activation of the ETS1 transcription factor by ERK (mutated *TERT* promoter bears ETS-binding sites) [68]. Epigenetic alterations may also contribute to altered MAPK signaling in melanoma, as observed with *miR-524-5p*, an miRNA that targets *BRAF* and *ERK2* transcripts and is down-regulated in melanoma [69]. On the other hand, other miRNAs like *miR-340* can function as tumor suppressor in melanoma by targetting distinct components of the RAS-RAF-MAPK signaling cascade [70].

### MAPK pathway as a therapeutic lead for melanoma treatment

Several compounds have been designed as therapeutic agents targetting the MAPK pathway, and these are in clinical trials and/or already approved for therapy. Specifically, Ras, Raf, MEK, and ERK have been the *bona fide* targets to be inhibited in many cancer treatments.

Sorafenib is a multi-tyrosine kinase inhibitor whose targets include B-Raf, platelet-derived growth factor receptor (PDGFR), and vascular endothelial growth factor receptor (Figure 1B). It was the first B-Raf inhibitor to reach clinical trials and, later, approved for renal cancer treatment [71], but failed to show antitumor activity in advanced melanoma when administered as single agent [72] or in combination with other therapeutic drugs [73].

In recent years, new potent specific BRAF^V600E^ inhibitors have been developed, including dabrafenib (GSK2118436) and vemurafenib (PLX4032]. Vemurafenib was the first B-Raf inhibitor approved by the U.S. Food and Drug Administration (FDA) for melanoma treatment [74] and both show good response and survival rates in metastatic melanoma [75,76]. Dabrafenib and vemurafenib associations are currently being studied in patients with unresectable and metastatic BRAF^V600E^ melanoma (NCT02967692, NCT01657591). CI-1040 (PD184352), a potent MEK inhibitor, progressed to clinical trials but presented adverse effects in ~60% of the patients [77]. Further tests revealed poor antitumor activity and low bioavailability of CI-1040 [78]. PD0325901, a second-generation analog of CI-1040, provided better results but, ultimately, showed higher toxicity [79]. Also, the second-generation MEK inhibitor selumetinib (AZD6244) failed to achieve progression-free survival (PFS) in melanoma [80], despite its antitumor activity. New generation of MEK inhibitors, such as trametinib (GSK1120212) and MEK162, have shown promising results in *BRAF* mutated melanoma [81] and are being tested in combinations in phase III clinical trials (NCT02967692 and NCT01909453).

Despite the efforts and success of MAPK inhibitors in cancer treatment, many patients with advanced melanoma still develop resistance to this chemotherapy. As a result, many MAPK inhibitors tend to be used in combination with other chemotherapeutic agents or even after cancer surgery. The mechanisms of resistance can be: (i) MAPK-dependent, when secondary alterations in MAPK pathway take place, such as amplification or splicing of *BRAF* [82], acquisition of additional activating mutations in *RAS, MEK1/2, ERK1/2*, or Raf isoform switching [83]; or (ii) MAPK-independent, such as altered RTK activity [84,85] and rescue of signaling by different ligands. Another mechanism of resistance to BRAF inhibition relates to the induction of programmed death-ligand 1 (PD-L1) expression, which transmits anergy signals to effector T cells, leading to suppression of an immune response [86]. Therefore, programmed cell death protein 1 (PD-1) receptor has been elected a therapeutic target, and antibodies against PD-1 such as PDR001 are being tested in melanoma clinical trials (NCT02807844 and NCT02404441).

## Phosphoinositide 3-kinase-AKT pathway

The phosphoinositide 3-kinases (PI3Ks) compose a family of lipid kinases with regulatory roles in a broad range of cellular mechanisms. PI3Ks are activated by diverse stimuli and/or stress-related insults, and are involved in a number of biological processes including cell survival, cell growth, differentiation, proliferation, transcription, and translation [87,88]. This pathway transduces signals from a variety of growth factors and cytokines, being the major downstream effector of RTKs and G-protein-coupled receptors (GPCRs) [87]. Thus, PI3K-Akt has been associated with the development of various diseases including diabetes mellitus, autoimmunity, inflammation, and cancer [87,89].

Three different classes of PI3Ks have been identified in mammals. PI3K Class I, the most well-characterized and also considered the main activator of the classical PI3K-Akt pathway, is composed by heterodimers containing a regulatory subunit (p85) and a catalytic subunit (p110]. This class is subdivided into class IA (p110α, p110β, and p110δ) and class IB (p110γ, and its regulatory subunit may be p101, p84 or p87]. Class IA enzymes can be activated by RTKs, GPCRs and certain oncogenes such as the small protein Ras, while the class IB enzymes are exclusively activated by GPCRs. The PI3K class II includes three different enzymes (PI3KC2α, PI3KC2β, and PI3KC2γ), consisting of a single catalytic subunit which can be activated by RTKs, cytokine receptors, and integrins. Still, the class II enzymes remain as the most enigmatic amongst all PI3K classes. The PI3K class III is largely recognized by its unique member, named vacuolar protein sorting associated 34 (Vps34 - also known as PI3KC3), which is also the only PI3K expressed in all eukaryotes. Vps34 is mainly a regulator of autophagy, implicated in integrating cellular responses to changes on nutritional status [87,90].

**Figure 2:**
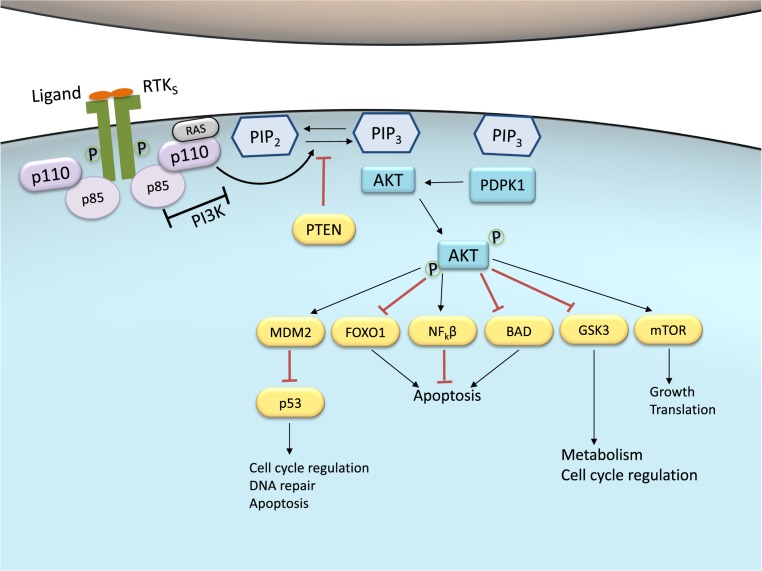
PI3K-Akt pathway activity in normal cells/tissues Under basal conditions, ligand binding to the RTK promotes receptor phosphorylation and activation. Activated RTK recruits the PI3K to the plasma membrane. Class IA PI3Ks are represented and may also be activated by GPCR and the small G protein Ras. These PI3Ks consist of a regulatory subunit (p85) and a catalytic subunit (p110). The activated p110 portion converts PIP_2_ into PIP_3_ at the membrane. PIP_3_ are docking sites for the serine-threonine protein kinase Akt, a crucial node in this pathway. Akt is phosphorylated and activated by putative PDPK1. p-Akt may elicit a wide range of downstream signaling targets that regulate cell cycle, apoptosis, DNA repair, glucose metabolism, growth and translation. PTEN dephosphorylates PIP_3_ back to PIP_2_ and therefore is the main negative regulator of the pathway, inhibiting Akt activation. Abbreviation: PDPK1, 3-phosphoinositide-dependent protein kinase 1.

Upon stimulation, the p85 subunit of PI3K interacts with the intracellular portion of the activated receptor. This interaction allows the activation of the catalytic subunit which, in turn, may associate with lipids in the membrane (Figure 2). Activated PI3K leads to the formation of phosphatidylinositol-3,4,5-trisphosphate (PIP_3_) by phosphorylating phosphatidylinositol-4,5-diphosphate (PIP_2_) in the plasma membrane. PIP_3_ constitutes a docking site for proteins that contain a Pleckstrin homology (PH) domain and also act as a potent second messenger, essential for the recruitment of the serine-threonine protein kinase Akt to the plasma membrane. Akt is a crucial node in this signaling pathway, transmitting signals by phosphorylating different downstream effector targets [87,90].

Akt, also referred to as protein kinase B (PKB), is expressed as three different isoforms: Akt1, Akt2, and Akt3 [91]. These isoforms are encoded by different genes but share similar protein structures: a PH domain, a central serine-threonine catalytic domain, and a regulatory domain. Akt normally exists in the cytosol in an inactive conformation and, when translocated to the plasma membrane upon PI3K activation, suffers a conformational change that exposes two residues for phosphorylation by 3-phosphoinositide-dependent protein kinase 1 (PDPK1) and 2 (PDPK2): Thr^308^ and Ser^473^, respectively. Once Akt is phosphorylated at both residues and fully activated, it turns a major downstream effector of PI3K pathway, inhibiting or activating a variety of targets (Figure 2) and, therefore, regulating important cellular behaviors such as apoptosis, DNA repair, cell cycle, glucose metabolism, cell growth, motility, invasion, and angiogenesis [87,90].

The main target of Akt is mammalian target of rapamycin (mTOR), which has a central role in PI3K-Akt pathway and cancer disease [87]. Regularly, mTOR plays a crucial part in the regulation of cell growth and proliferation by monitoring nutrient availability, cellular energy, oxygen levels, and mitogenic signals [87,92]. mTOR exerts its function by forming two different complexes: mTOR protein complex 1 (mTORC1) and complex 2 (mTORC2). The first complex (mTORC1), largely involved in ribosomal biogenesis and protein synthesis, is composed by the mTOR catalytic subunit, the regulatory associated protein regulatory associated protein of mTOR (RAPTOR), protein mLST8 and proline-rich Akt substrate 40 kDa (PRAS40) [87,92]. The mTORC2 complex consists of rapamycin-insensitive companion of mTOR (RICTOR), mammalian stress-activated protein kinase interacting protein 1 (MSIN1) and mLST8, and is involved in cytoskeleton remodeling, cell survival, and glucose metabolism [87]. Uncontrolled activation of mTOR and its complexes mTORC1 and mTORC2 may favor tumor development, promoting cell growth and protein synthesis [87].

Like many other cellular cascades, PI3K-Akt signaling has regulators to timely control any persistent and long-term activation. A main negative regulator of PI3K is the tumor suppressor PTEN, which antagonizes the PI3K activity by its intrinsic lipid phosphatase activity, converting PIP_3_ back into PIP_2_. Loss of PTEN results in constitutive activation of Akt, and it has been largely associated with tumor development in malignant melanoma [93].

### Activity of PI3K-Akt pathway in melanoma

As previously stated, melanoma is a complex genetic disease whose initiation and progression involves mutations and impaired function in different cell signaling pathways. The PI3K signaling cascade has been shown to be up-regulated in different types of cancer, including melanoma, to protect the tumor tissue from stress conditions that could lead to cell death and consequent regression [94,95]. Oncogenic events that activate PI3K-Akt may include mutations or copy number variations in certain pathway components. Mutations in cross-talk components, such as Ras protein family, and mutated or amplified RTK expression may also hyperactivate PI3K-Akt pathway [96].

Melanocyte function relies on the activation of the RTK c-Kit and its ligand stem cell factor (STC) within the epidermis [5,6]. c-Kit can activate the PI3K-Akt pathway, thus being fundamental to the development of normal skin, controlling melanocyte permanent survival, proliferation, and migration [97]. c-Kit and STC also regulate melanocyte survival in adult normal skin [5,6].

The physiological role of PI3K-Akt in melanocytes is strictly regulated. A pathway malfunction may lead to cell cycle impairment, favoring cancer development by losing the requirement of c-Kit activation for cell survival and proliferation [87,98]. Indeed, mutations and amplifications of *KIT* are detected in 10–15% of acral melanomas, 20–30% of mucosal melanomas, and 1% of other cutaneous tumors [99]. In addition, c-Kit activity is exacerbated in human epidermis exposed to UV light [100].

Mutations in *PI3K* genes are relatively rare in melanoma, with a frequency of ~3% in metastatic melanoma [101]. Akt is also mutated at low extent in melanoma [8,102]. However, Akt (mainly Akt3) is overexpressed in 43–60% of non-familial melanomas, and it has been implicated in disease progression, metastasis, and development of drug resistance [95,103]. Consistently, patient survival is inversely correlated to activated Akt levels [104]. Nevertheless, a more in-depth understanding of Akt functions in melanoma is still warranted [95].

Mutations in *MTOR* genes have not been fully investigated, although they are apparently frequent in melanoma patients. A recent study, composed of 412 melanoma samples, has shown that 10.4% of these melanoma cases presented somatic *MTOR* mutations [105]. Interestingly, melanoma patients with *MTOR* mutations often have shorter survival rates, suggesting that these mutations may lead to worse prognosis of the disease [105]. The mTORC2 component RICTOR has also been found to be overexpressed in melanoma, where it appears to stimulate clonogenicity [92]. *AKT* overexpression also increases mTOR activity, leading to unbalanced mTOR functions which are normally regulated by nutritional bioavailability, necessary for cell proliferation [106].

**Figure 3:**
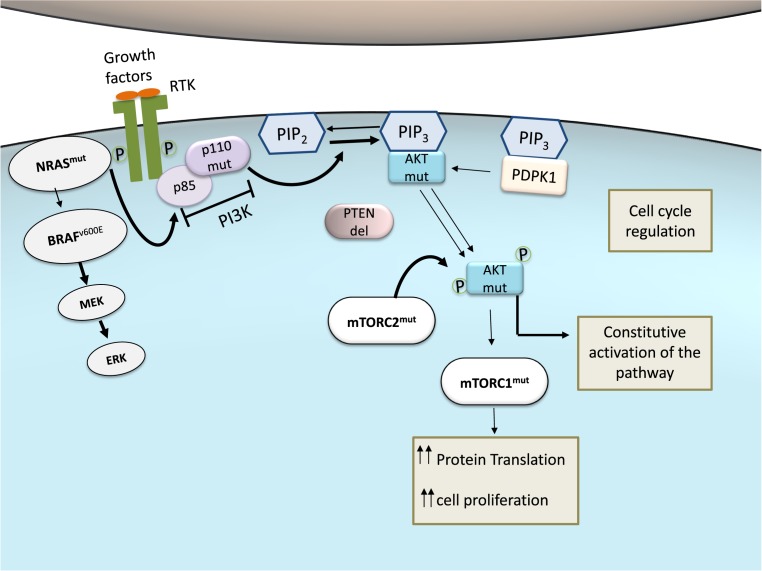
PI3K-Akt pathway activity in melanoma Malignant transformation in melanocytes can require combinations of frequently seen genetic defects, which lead to melanoma development. These mutants often elicit Akt over-activation, leading to constitutive pathway activation (indicated by thicker arrows). Constitutive activation of PI3K-Akt signaling impairs cell cycle regulation, favoring tumor initiation and progression. Mutations can be mutually exclusive or coexist. For example, mutated NRAS (NRAS^mut^) and BRAF (BRAF^V600E^), from Ras-Raf-MEK-ERK pathway, rarely occur in the same melanoma cell, whereas BRAF^V600E^ and PTEN function loss (PTEN^del^) appears in 20% of melanomas. NRAS^mut^, a well-described mutation in melanoma cells, may also lead to hyperactivation of the PI3K-Akt pathway. PI3K mutation itself (p110^mut^) is a rare condition. mTORC1^mut^ increases protein translation and cell proliferation, favoring melanoma development. Also, mTORC2^mut^ enhances Akt activation, collaborating with the maintenance of constitutive activation of PI3K-Akt pathway in cancer.

In addition to point mutations, PI3K-Akt signaling may also be hyperactivated in melanoma due to loss of activity of the negative regulator PTEN (Figure 3). PTEN function is lost in 10–30% of cutaneous melanomas, leading to constitutive activation of PI3K pathway [107]. Interestingly, *PTEN* gene mutations and deletions are mutually exclusive in melanoma with activating *N-RAS* mutation [108]. On the other hand, ~20% of melanomas with PTEN loss-of-function also have *BRAF^V600E^* mutations [109,110]. Despite the unclear significance of these observations, current studies suggest that these may increase the metastatic potential of melanoma [111].

The many possible regulatory mechanisms and the variety of components of PI3K-Akt pathway illustrate its own complexity. To inhibit this pathway in melanocytes, it has been shown that the inactivation of more than one single pathway component is generally necessary (such as PI3K and mTOR complexes simultaneously) [92]. Otherwise, the pathway can be reactivated at short time, even if PI3K is still inhibited. This suggests the existence of additional feedback mechanisms downstream of PI3K, dependent on mTOR complexes [92].

### PI3K-Akt pathway as a therapeutic lead for melanoma treatment

The development of drugs targetting the PI3K-Akt pathway in melanoma looks promising, but still faces numerous challenges. Pharmacologically, there has been intense development of inhibitors against PI3K, Akt, mTORC1, dual PI3K/mTOR, and dual mTORC1/mTORC2 [112]. Perifosine, for instance, is a synthetic oral inhibitor of Akt that has been tested in pre-clinical and clinical trials against many types of cancer, including melanoma. This drug inhibits the translocation of Akt to the plasma membrane, blocking its activation. However, current tests show several toxic effects and no relevant benefits toward patient survival or disease regression [113].

PI3K-Akt inhibitors are not still available for clinical treatment, due to a number of reasons that impair their development, which include: (i) these drugs lack consistency on their affinity toward different isoforms of signaling proteins; (ii) different types of melanoma have different optimal target proteins, depending on the mutated gene; (iii) the development of targetted therapies relies on specific patient portfolios; (iv) lack of appropriate markers to assess the pharmacodynamics of these agents; (v) existence of feedback compensatory loops within and in between cell signaling pathways that control cell proliferation and apoptosis [96,114]. In fact, this last reasoning may explain the lack of clinical activity in melanoma for some single-agent mTORC1 inhibitors, such as CCI-779/Temsirolimus (NCT00022464), a rapamycin derivate [115]. Rapamycin and its derivatives are well-known mTOR experimental inhibitors [116].

While many studies support the causal role of activating *BRAF* and *NRAS* mutations in melanoma progression, opposing data suggest that the contribution of PI3K-Akt pathway to this disease may be variable [117]. In this sense, PI3K-Akt inhibitors might be used in combination with other agents that induce apoptosis [27], but not as single agents. Recently, Akt3 and Wee1 (a protein involved in cell cycle regulation) have been defined as putative and simultaneous targets to synergistically inhibit melanoma *in vitro* [118]. Moreover, despite the limited experimentation, a Chinese herb named *Alocasia cucullata* has recently shown activity to up-regulate expression levels of *PTEN* and decrease phosphorylation of PI3K and Akt *in vivo* and *in vitro*, leading to an antimelanoma effect [28]. Clinically, a PI3K-inhibitor (GSK2636771) is being tested, itself or in combination with PD-1 inhibitor Pembrolizumab, in refractory melanoma patients with PTEN loss (NCT03131908).

## WNT pathway

In humans, Wnt proteins compose a family of 19 highly conserved and secreted glycoproteins that act through a variety of receptors to stimulate distinct intracellular subpathways [119]. These pathways are typically involved in embryonic development, cell growth, migration, and differentiation [119]. The Wnt signaling is also required throughout life for the homeostatic balance of quickly self-renewing tissues, such as intestinal crypts and hair follicles, as well as for the regulation of bone mass [120]. Therefore, impairment of Wnt signaling cascade results in severe embryonic malformations as well as in a broad range of other pathological conditions [119,121].

**Figure 4:**
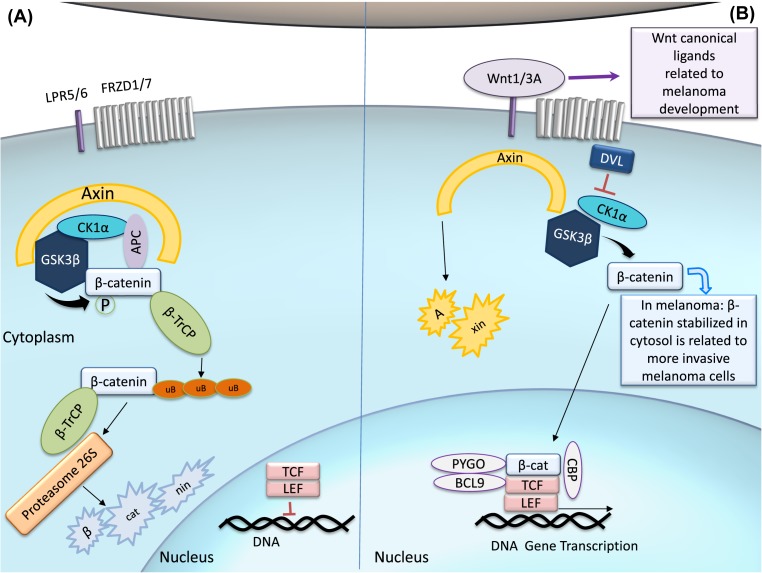
Canonical Wnt signaling cascade (**A**) When there is an absence of Wnt ligand, the cascade is ‘off’ and β-catenin is recruited to the APC and axin complex. This allows β-catenin to be phosphorylated by CK1α and GSK3β, and then subsequently ubiquitylated by β-TrCP. Following ubiquitylation, β-catenin is directed to the proteasome to be degraded. Low levels of cytoplasmic β-catenin repress gene transcription of Wnt targets by LEC and TCF. (**B**) In the canonical pathway, Wnt ligands binds to the FRDZ receptor and LPR5/6, which are phosphorylated by CK1α and GSK3β. It evokes DVL to interact with p-FRDZ, leading to the inactivation of the destruction complex, preventing β-catenin degradation. Stabilized, β-catenin translocate into the nucleus and increases gene transcription by forming a complex with TCF, LEF and the co-factors PYGO, BCL9, and CBP. In melanoma cells, WNT1 and WNT3A are the major ligands responsible for the constant activation of Wnt canonical pathway, leading to β-catenin stabilization in the cytosol, which favors melanoma progression. Abbreviations: APC, adenomatous polyposis coli; CBP, cAMP response element-binding protein; CK1α, casein kinase 1α, DVL, dishevelled; GSK3β, glycogen synthase kinase 3β; LEF, lymphoid enhanced transcription factor; PYGO, Pygopus; TCF, T-cell transcription factor; β-TrCP, β-transducin repeat-containing protein.

Wnt ligand-eliciting pathways may be subdivided into two main categories: the canonical pathway, which includes the intracellular transcriptional co-activator β-catenin as a central component; and alternative cascades that lack the participation of β-catenin [122]. The canonical Wnt pathway may be activated by Wnt proteins, such as WNT1/WNT3A (Figure 4), through the binding to Frizzled receptors (FRZD1-7) and co-receptors, lipoprotein receptor (LRP) 5 (LRP5) and LRP6 [123]. FRZDs are seven transmembrane domain (TM) cell surface receptors that belong phylogenetically to the large family of GPCRs [124]. The LRP5/6 co-receptors stimulate the intracellular signaling to further regulate β-catenin stability and context-dependent transcription [125].

Upon stimulation of canonical Wnt signaling, the cytosolic downstream mediator scaffold protein Dishevelled (DVL) becomes activated and prevents β-catenin from degradation by inhibition of glycogen synthase kinase 3β (GSK3β) [126]. The blocking of β-catenin degradation leads to its stabilization in the cytosol, allowing its translocation in the nucleus and the association with transcription factors, such as lymphoid enhanced transcription factor (LEF) and T-cell transcription factor (TCF) [127]. Upon binding of β-catenin to LEF/TCF, these factors become transcriptional activators by (i) displacing their repressor Groucho and (ii) being able to recruit other transcriptional co-activators, such as cAMP response element-binding protein (CBP) and B-cell lymphoma 9 (BLC9) [123]. In this manner, β-catenin-containing complexes control the expression of several Wnt target genes, including *c-MYC* and *CCND1* [122,128].

In the absence of Wnt ligands, the activity of β-catenin is strictly controlled by the action of a degradation complex, composed by GSK3β, casein kinase 1α (CK1α), and the scaffolding protein Axin. These proteins contain a regulator of G-protein signaling (RGS) domain that interacts with adenomatous polyposis coli (APC), a large multifunctional scaffolding protein that binds β-catenin itself. This complex is responsible for promoting the phosphorylation of β-catenin by GSK3β and CK1α [123]. After being phosphorylated, β-catenin may be ubiquitylated by β-transducin repeat-containing protein (β-TrCP), which leads to its proteasome degradation [129]. β-catenin processing can be further modulated by protein phosphatase 2A (PP2A) and protein phosphatase 1 (PP1), which are ubiquitously expressed serine–threonine phosphatases that interfere with the complex function by dephosphorylating β-catenin and then disengaging its destruction [130,131].

Some Wnt ligands have also been shown to interact with other transmembrane receptors types, including RTKs and RTK-like orphan receptor (RoR) family RoR2 [132]. They can act independently of LRP5/6 and thus may activate β-catenin-independent pathways. This activation can lead to changes in cell movement and polarity, and even antagonize the β-catenin pathway [120,125]. FRZDs have also been shown to respond to Wnt proteins in the absence of LRP5/6, being able to elicit β-catenin-independent pathways [125]. Non-canonical Wnt pathways are diverse and, in most of the cases, still poorly defined [133].

### Activity of Wnt pathway in melanoma

The canonical Wnt pathway is responsible for controlling, to some extent, cancer cell metabolism [122]. In melanoma, Wnt/β-catenin-mediated metabolic reprogramming of the cancer cells can directly affect vessel density, and it is strongly linked to tumor angiogenesis by directly regulating the expression of pro-angiogenic growth factors [134]. Still, in this context, Wnt regulation is poorly understood [122].

As previously stated, the canonical Wnt pathway controls the expression of *c-MYC*, a well-characterized proto-oncogene [122]. c-MYC functions as a transcription factor by binding to enhancer box sequences of a number of target genes, many of which are involved in cell cycle control, including cyclins, CDKs and CDK inhibitors [135]. Metabolic events including glycolysis, nucleotide synthesis, lipid synthesis, glutaminolysis, and mitochondrial bioenergetics are deeply controlled by Myc-driven transcriptional regulation in cancer cells, all of which are essential for biomass accumulation and genome replication in rapidly dividing cells [136]. In fact, aerobic glycolysis has been associated with the function of WNT5A in melanoma cells, and this activity has been related to an increase in active Akt signaling and high lactate dehydrogenase (LDH) levels in the serum [137]. Melanoma cells primarily use glycolysis as an energy source for cell motility and, in this sense, WNT5A acts as a positive modulator by promoting both migration and aerobic glycolysis [137].

The canonical Wnt proteins WNT1 and WNT3A are also known to be critical for melanocyte transformation (Figure 4B) and, consequently, avoidance of cellular senescence [138]. Melanoma tumors frequently exhibit constitutive activation of the Wnt/β-catenin pathway, presumably to induce cell proliferation, but conflicting studies on the role of β-catenin in melanoma metastasis have been reported [119]. Paradoxically, melanoma patients with higher β-catenin levels have shown a better disease prognosis [139], implying that the mechanisms that drive melanomagenesis are distinct from those that dictate its outcome. Nevertheless, in defined genetic contexts, such as *in vivo* melanoma models where *BRAF* is mutated, PTEN is usually deleted and β-catenin is then stabilized, leading to an increase in metastatic events [140]. Moreover, at different stages of melanoma progression, the expression and distribution of Wnt receptor(s)/co-receptor(s) can also define the fate of the signaling activation [120].

The canonical Wnt signaling can co-operate with MAPK signaling to regulate the expression and activity of the master transcription regulator MITF, which is associated with melanoma cell proliferation [141]. The effects of Wnt/β-catenin and MITF signaling is apparently context-dependent, where activating mutations and the intracellular localization of β-catenin may play a role [141]. Intracellular localization of β-catenin also correlates with the association of elements in the cellular phenotype, since membrane-associated β-catenin has been found in proliferative cells, while cytosolic β-catenin has been more detected in invasive cells [142].

Melanoma cells also present *ROR2* up-regulation, which promotes invasion, metastasis, and therapeutic resistance through distinct intracellular signaling cascades, including β-catenin-independent cascades [143]. WNT5A/ROR2 signaling induces recruitment and activation of Src kinase activity to promote metastasis [144]. Moreover, WNT5A induces depalmitoylation of melanoma cell adhesion molecule (MCAM) and, subsequently, polarizes localization of MCAM and CD44 (another cell adhesion molecule) to promote directional movement and invasion of melanoma cells [145]. The non-canonical Wnt signaling pathway, mediated by WNT5A, has also been associated with increasing metastatic potential of melanoma cells and respective tumor grades [146].

A number of other proteins, important for the positive modulation of the Wnt pathway, have recently been associated with cancer development, such as members of the R-spondin ligand family that bind to the leucine-rich repeat containing GPCRs (LGR) 4–6 [147]. In the absence of R-spondin binding, the two homologs E3 ubiquitin ligases ZNRF3/RNF43 target FRZD for lysosomal degradation, which is dependent on DVL. Upon LGR activation by R-spondin, this complex inhibits the activity of ZNRF3/RNF43 and leads to the accumulation of FRZD receptors on the cell surface. ZNRF3/RNF43 work as negative regulators of Wnt signaling since they are transcriptional targets of this pathway [121,148]. The discovery of the R-spondin/LGR5/RNF43 signaling module and its genetic alterations in cancer represents a breakthrough in this area. However, the frequency of RNF43 mutations in melanoma is lower than 2%, and the mutation rate of LGF5 in cutaneous melanoma still requires further investigation [121,149].

### Wnt pathway as a therapeutic lead for melanoma treatment

Wnt signaling components, mainly including FRZDs and DVL, have been considered as targets for cancer treatment using small molecule inhibitors [150]. Specific DVL inhibitors have been developed using protein–protein interaction screens and design-based algorithms [151,152].

Some natural compounds, such as chalcones, derricin, and derricidin, are newly discovered inhibitors of the Wnt signaling pathway. Their inhibitory function is characterized by reduced amounts of nuclear β-catenin in treated cells. Elucidating the mechanism of action of these drugs is warranted, since they may offer advantages on lowering the risk of mutagenesis (due to their limited interaction with the DNA) [153].

Extra- and intracellular components of the Wnt pathway, including axin, APC, and β-catenin, are frequently associated with a variety of human cancers, and therefore, antibody-based therapies have also been proposed to target-specific pathway components [154]. OMP-18R5 (vantictumab) is an antibody that interferes in the binding of Wnt ligands to human FRZDs. It has demonstrated activity in inhibiting the growth of a range of human tumor xenograft models, and exhibits synergistic activity when co-treated with standard chemotherapeutic agents [155]. Similarly, antibody-based blockage of WNT5 induces inhibition of protein kinase C (PKC) activity and decrease in cellular invasion [141]. Indeed, WNT5A has shown an important role in the invasive behavior of melanoma cells, since constitutive *WNT5A* overexpression is able to induce actin reorganization and increased cell adhesion [156]. Moreover, a phase I study (NCT01351103) with an oral drug LGK974, which blocks Wnt signaling, is being conducted to find safe doses for melanoma and others solid malignancies.

Another potential therapeutic strategy targetting Wnt signaling relates to the development of specific peptides that mimic Wnt proteins and, therefore, lead to receptor inhibition. For instance, UM206 is a small peptide derived from regions of high homology between WNT3A and WNT5A that has been demonstrated an antagonist effect on FRZDs, therefore blocking downstream Wnt signaling [157]. This strategy has been applied in other diseases, such as heart failure [157] and hepatocellular carcinoma [158], and may be developed for melanoma treatment in the near future.

## Nuclear factor κ-light-chain-enhancer of activated B cell pathway

The nuclear factor κ-light-chain-enhancer of activated B cell (NF-κB) pathway was first described as a gene expression regulator in B lymphocytes [159] and, therefore, initially considered as a positive modulator of inflammation and immune response. NF-κB signaling is required during early development [160,161] and its molecules are evolutionarily conserved [162], implying a broader role in other physiological activities and conditions [163]. In fact, NF-κB activation is triggered by a diverse range of stimuli, such as cytokines (tumor necrosis factor α (TNF-α), interleukin 1 (IL-1), growth factors, bacterial lipopolysaccharide (LPS), ROS, UV, and ionizing radiation [164,165], which drives the transcription of a variety of target genes, including interleukins, chemokines, proteases, and apoptosis-related genes, leading to a plethora of outcomes such as stress response, immunity, cell proliferation, and apoptosis. By being involved in the induction of proliferation genes, inhibition of apoptosis, and regulation of immune and inflammatory responses [166], NF-κB has been largely investigated in the context of tumor progression and immune evasion. In fact, NF-κB has been implicated in cancer initiation, promotion, metastasis, resistance to treatment, and may be also implicated in carcinogenesis by regulating genome stability [167]. On the other hand, TNF-α induced by viral infection promotes cytolysis in fibrosarcoma cells [168] implying that, upon different stimuli, TNF-α induces apoptosis.

NF-κB comprises a family of transcription factors that act as homo- or heterodimers and, upon activation, translocate into the nucleus where they interact with specific tandem DNA structures (κB enhancers) that modulate target gene expression [169]. In the absence of stimulus, NF-κB dimers are typically sequestered and inactivated in the cytoplasm by inhibitors of NF-κB inhibitor of NF-κB (IκB family). The IκB proteins, including IκBα, IκBβ, and IκBε, contain multiple ankyrin repeats in their structures which are responsible for their inhibitory activity [170]. The ankyrin repeats found on IκB proteins interact with the Rel homology domain (RHD) of Rel proteins, masking their nuclear localization signal (NLS) and therefore sequestering them in the cytosol [171].

In mammals, five NF-κB family members have been described: RelA (p65), RelB, c-Rel, p100/p52, and p105/p50 [170]. All of them share a conserved 300 amino acid N-terminal RHD, which harbors sequences that are crucial for DNA binding, dimerization, IκB interaction, and nuclear localization [172]. The Rel proteins (i.e. p65, RelB, and c-Rel) have a C-terminal transactivation domain (TAD) that enable interaction with different transcriptional regulators to further induce κB-responsive gene expression [171]. In contrast, due to an IκB-like structure at their C-terminus, p100 and p105 may serve both as precursors of NF-kB members p52 and p50, respectively, and also as inhibitors of NF-κB dimers [173].

**Figure 5:**
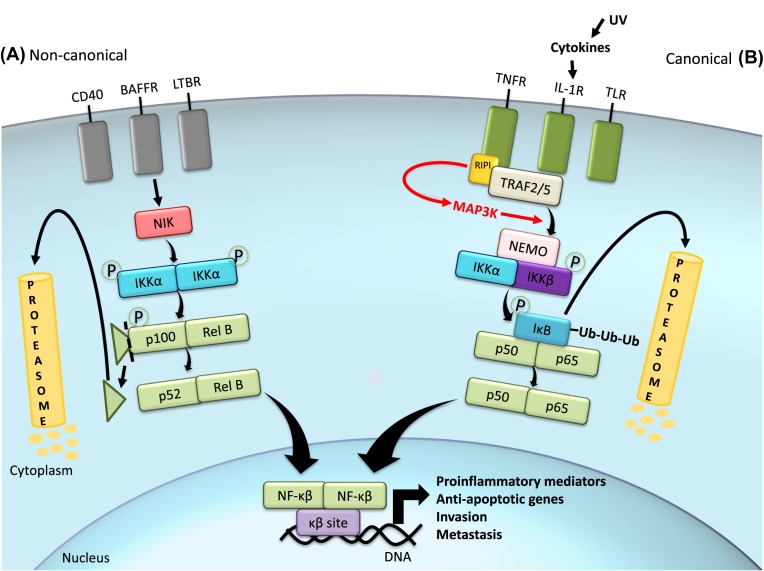
Canonical and non-canonical NF-κB signaling pathways The NF-κB cascade can be initiated by several types of receptors, which will trigger either the canonical or the non-canonical pathway. When the canonical pathway is initiated by receptors such as TNFR, the activated receptor interacts with associated proteins (TRAFs), which recruit RIP1 kinase to be phosphorylated by the receptor. This pathway culminates in the phosphorylation and activation of the IKK complex formed by scaffold protein NEMO, IKKα, and IKKβ. NF-κB dimers are conjugated with inhibitor IKB in the cytoplasm. IKK complex is responsible for phosphorylating IKB and therefore targetting it for degradation, releasing NF-κB to translocate into the nucleus and promote transcription of target genes by binding to the κB site in the DNA. The non-canonical pathway is initiated by other receptors, which relies on the activation of IKKα homodimer by the NF-κB inducing kinase (NIK), assisted by TRAF. p100:RelB, the most common NF-κB dimer in this pathway, is phosphorylated on the p100 subunit, which will be cleaved in its inhibitory domain and release active p52:RelB dimer to exert its nuclear functions. In melanoma, UV light exposure is one of the factors that might activate NF-κB by inducing cytokines that signals through this pathway to induce a pro-inflammatory response. Sustained activation of NF-κB also promotes the expression of anti-apoptotic genes, as well as invasion- and metastasis-related genes.

The p50:RelA(p65) dimer is the prototypical dimer in the canonical (or classical) pathway [174], but each combination of dimers stimulates different responses by inducing the expression of specific target genes. The canonical NF-κB pathway is mainly activated by TNF-α, IL-1 and Toll-like receptors (TNF-α receptor (TNFR), IL-1 receptor (IL-1R) and TLR, respectively), and culminates in the activation of the IκB kinase (IKKα)/IKKβ/NF-κB essential modulator (NEMO) complex by phosphorylation events (Figure 5). For instance, TNFR1 possesses auto-phosphorylation capacity that leads to receptor activation [175]. Once activated, TNFR interacts with TNFR-associated factors (TRAFs) 2 and 5, which recruit receptor interacting protein 1 (RIP1) kinase to be phosphorylated by the receptor [175]. RIP1 can further activate p38 MAPK and MAP3K MEKK3, which promote phosphorylation of the IKK complex and signal propagation [176]. The IKK complex comprises two kinases (IKKα and IKKβ) and a regulatory and scaffold protein called NEMO (or IKKγ). The activated IKK complex phosphorylates the NF-κB-bound IκB (at Ser^32^ and Ser^36^ on IκBα, and Ser^19^ and Ser^23^ on IκBβ), targetting IκB for further polyubiquitination and proteasome-dependent degradation.

On the other hand, the non-canonical pathway, which is activated by lymphotoxin-β (LTβ), B-cell activating factor (BAFF) and cluster of differentiation 40 ligand (CD40L), relies on the activation of IKKα homodimer by the NF-κB inducing kinase (NIK), assisted by TRAF [177]. In fact, the IKK subunits may assemble in distinct conformations in the canonical and non-canonical NF-κB pathway. IKKα phosphorylates the C-terminus of p100, leading to a proteasome-dependent degradation of its inhibitory domain and then producing active p52. Then, mature p52 dimerizes with Rel proteins (p52:RelB is known as a prototypical non-canonical dimer) and then translocates into the nucleus to regulate selective immune responses, such as lymphoid organogenesis and B-cell maturation and survival [178].

### Activity of NF-κB pathway in melanoma

NF-κB participates in epidermal homeostasis [179]. UV irradiation promotes cytokine production in skin cells [180] which, at short-term exposure, can induce an inflammatory response by releasing interleukins, granulocyte colony stimulation factor (G-CSF), macrophage colony stimulating factor (M-CSF), transforming growth factor α (TGF-α), and others [181]. Many of these cytokines have NF-κB as their downstream target/effector or may even have their expression modulated by NF-κB itself, like the *IL-1, TNF-α*, and *IL-6* genes. Therefore, sustained activity of NF-κB may lead to exacerbated expression of pro-inflammatory mediators, therefore causing tissue injuries that may evolve into organ dysfunction and eventually cancer [182].

In melanoma, NF-κB activity can lead to the induction of chemokines such as C–X–C motif chemokine ligand 1 (CXCL1), which constitutes a feed-forward mechanism by activating the IKK complex [183]. Loss of E-cadherin, a common feature of malignant transformation, has been associated with up-regulation of NF-κB in melanoma cell lines [184]. NF-κB largely contributes to melanoma through its function in regulating survival and apoptosis [185]. An imbalance between pro- and anti-apoptotic genes favoring the latter has been demonstrated in melanoma cells by increased expression of RelA, p50, anti-apoptotic molecules (such as inhibitor of apoptosis (IAP) and caspase-8 inhibitory protein (FLIP)), and decreased expression of c-Rel, a pro-apoptotic regulator [186].

Deregulation of NF-κB can occur at different levels of the pathway. Constitutive activation of IKK, resulting into continuous I*κ*B degradation and sustained increase in NF-*κ*B in the nucleus, has been observed in malignant melanoma [183]. NF-κB signaling has also been shown to be influenced by other pathways that are commonly altered in cancers, including melanoma. For instance, Akt can mediate IKKβ- and p38 kinase-dependent phosphorylation of RelA transactivation domain, which is required for its transcriptional activity [187]. Also, p50 phosphorylation, mediated by PI3K-Akt signaling, increases its DNA-binding capacity [188]. NF-κB signaling may also participate in cancer invasion and metastasis by inducing the expression of adhesion molecules, such as intercellular adhesion molecule-1 (ICAM-1) and vascular cell adhesion molecule-1 (VCAM-1), and metalloproteinases (MMPs) [189–191].

*TERT* promoter mutations are key drivers of telomerase reactivation in cancer and in melanoma in particular [192,193]. Single-residue mutations take place on two locations in the promoter [192,193] and create binding sites for ETS family of transcription factors, which require co-operation with other binding partners to exert their transcription activity. Non-canonical NF-κB subunit p52 is a binding partner of ETS and, together, they interact with mutated sites on *TERT* promoter and induce its activation [194]. Long-range chromatin interactions have been demonstrated between other binding partners and a region that is more than 300 kb in size, upstream of *TERT* promoter [195]. This is required for the interaction of key distal elements to the proximal promoter. Indeed, it is known that reactivated telomerase has both canonical and non-canonical activities which may drive melanoma progression [196–198].

### NF-κB pathway as a therapeutic lead for melanoma treatment

Despite its constitutive activation in melanoma, targetting NF-κB for cancer therapeutics has been challenging mostly due to its role in cytokine and chemokine production, therefore offering relative risks to the migration of immune cells and immune response. However, NF-κB inhibition can promote benefits by enhancing apoptosis, and this may be achieved by targetting cross-talk regulators within PI3K/Akt and MAPK pathways [199]. Administration of non-steroidal anti-inflammatory drugs (NSAIDs), in melanoma *in vivo* models, can inhibit tumor growth without causing detrimental effects [200]. Therefore, the use of NSAIDs have been considered as a potential therapeutic approach in melanoma. Proteasome inhibitors, such as bortezomib, have been used to inhibit NF-κB by impeding IκB degradation. These inhibitors are shown to induce enhanced apoptosis in multiple myeloma [201] and decreased cell proliferation in melanoma *in vitro* [202]. Bortezomib also induces reduction in tumor growth *in vivo* [202] but has shown significant toxicity after clinical trials [203,204].

Recent efforts have been directed to developing selective inhibitors of the NF-κB pathway. BMS-345541, a specific IKK inhibitor, has been shown to reduce constitutive IKK activity and induced apoptosis of melanoma cells [205]. The NBD (NEMO-binding domain) peptide can bind to NEMO and prevent its interaction with IKKα/β [206], which is crucial for IKK complex activity and activation of NF-κB. The use of NBD peptides can promote growth arrest and apoptosis in human melanoma cell lines [207], but neither NBD peptides nor BMS-345541 have reached clinical trials yet.

## Janus kinase-signal transducer and activator of transcription pathway

The Janus kinase (JAK)-signal transducer and activator of transcription (STAT) signaling pathway is involved in various biological events in mammals, such as cell proliferation, differentiation, migration and apoptosis, exerting fundamental role in multiple physiological process, which range from mammary gland development, lactation, adipogenesis to inflammation and other immunological responses including pathogen resistance [208,209]. Evolutionarily, it is a well-conserved pathway amongst several living species, including humans, flies, and worms, apparently resulting from early adaptation mechanisms to facilitate intercellular communication [209,210].

In mammals, the JAK family is constituted by four members: JAK1, JAK2, JAK 3, and TYK2 [211]. All JAKs share a tyrosine kinase domain that relates to their trans-phosphorylation activity. The receptors that trigger JAK-STAT signaling can be activated by several ligands, including cytokines, growth factors, and hormones. The ligand binding to the JAK-associated receptors leads to their dimerization, bringing JAK molecules into close proximity and allowing them to phosphorylate each other. Unlike other types of receptors, these cytokine receptors do not hold an intrinsic kinase activity, relying on JAK’s phosphorylation activity to cover this function. Moreover, through its kinase activity, JAKs also phosphorylate the receptor itself and their major target: the STATs [208,209].

STATs are cytoplasmic transcription factors that remain latent until tyrosine phosphorylation (and consequent activation) by JAKs. In mammals, there are seven different types of STATs: STAT1–4, STA5A, STAT5B, and STAT6 [212]. Once activated, through interaction of conserved SH2 domains, STATs form homo- or heterodimers and then translocate to the nucleus. In the nucleus, the dimerized STATs are competent to bind DNA and to modulate specific sets of genes, activating or repressing their transcription [208,209].

Like other signaling pathways, there are a number of intracellular components that modulate this signaling cascade [209]. One of these components are the suppressor of cytokine signaling (SOCS) proteins [213]. In mammals, the SOCS family is composed by eight members (SOCS1–7 and CIS) which all act as negative regulators of JAKs [214]. Since *SOCS* expression is also stimulated by JAK-STAT activation, this feature constitutes a feedback-loop mechanism of signaling control [214]. There are three putative ways in which SOCS may down-regulate JAK-STAT signaling: (i) SOCS binds directly to JAKs and blocks their kinase activity; (ii) SOCS binds to phosphotyrosines along the receptors, preventing STATs and other signal transducers from docking and then getting phosphorylated; and (iii) SOCS promotes ubiquitination and consequent degradation of JAKs (and related receptors) by interacting with elongin BC complex and cullin 2 [208].

Another negative regulator of JAK-STAT signaling is the protein inhibitor of activated STAT (PIAS). Unlike SOCS, PIAS is not induced by JAK-STAT signals but, instead, it is constitutively expressed [215]. PIAS binds to activated STAT dimers and mostly blocks their nuclear translocation by mechanisms not fully understood [216]. JAK-STAT activity may also be down-regulated by protein tyrosine phosphatases, from which the most well-described is SHP-1. These phosphatases act by dephosphorylating JAKs and/or their receptors, terminating the signaling activation [208,216]. Noteworthy, the duration and intensity of the signal(s) vary according to a balance between positive and negative pathway regulators in a cell-specific manner [209].

**Figure 6:**
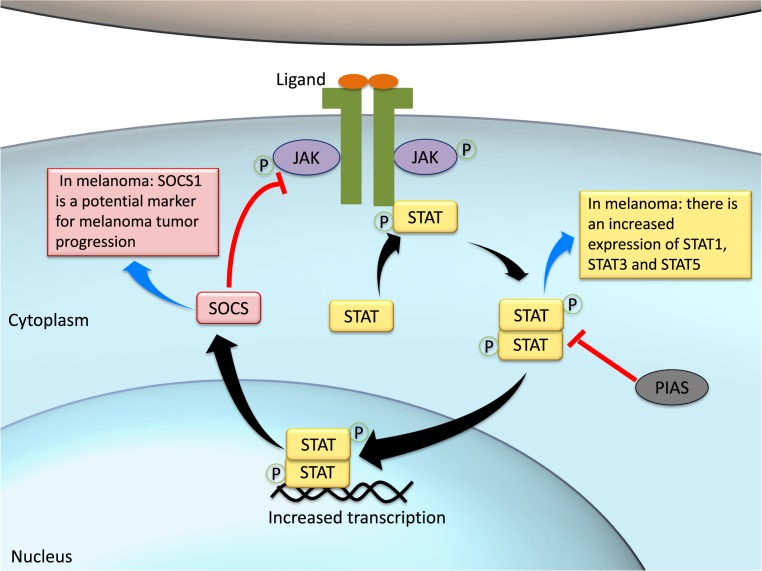
JAK-STAT pathway activation and its negative regulators Upon ligand stimulation, JAK-binding receptors aggregate in the plasmatic membrane forming dimers and bringing JAKs into close proximity. This allows JAKs to be trans-phosphorylated and further phosphorylate the receptor itself. The phosphorylated receptor is a docking site to STATs, which are also phosphorylated and activated by JAKs. Phosphorylated STATs (p-STATs) form dimers with other p-STATs, becoming active and able to translocate into the nucleus, where they stimulate gene transcription. The JAK-STAT pathway is then an effective way to communicate extracellular signals directly to the nucleus without the need of second messengers. This cascade may be regulated by SOCS, which blocks JAK kinase activity and also by PIAS that deactivate the STAT dimer. In melanoma context, there is a balanced overexpression of STAT1, STAT3, and STAT5. On the other hand, there is SOCS1 silencing in melanoma cells, which constitutes a potential biomarker for the disease.

It has been also described that non-classical JAK-STAT pathways may act by serine phosphorylation, acetylation, methylation, and sumoylation of diverse proteins, and controlling cellular epigenetic status [217]. Considering that JAK-STAT signaling plays a role in such a variety of processes, it is not surprising that disturbances of this pathway are involved with disease development, including melanomagenesis (Figure 6).

### Activity of JAK-STAT pathway in melanoma

It has been reported that JAK-STAT pathway may exert a pivotal role in numerous aspects of tumor development, including initiation, cell proliferation, differentiation, inhibition of apoptosis, enhanced invasiveness, immune surveillance, and angiogenesis [218,219]. This cascade is over-activated in some types of melanoma, where STAT3 seems to be crucial, triggering the transcription of anti-apoptotic and pro-survival genes [220,221]. STAT3 activity is typically increased in higher cell density, and this process is mediated by JAK activation [222]. Also, STAT3 promotes growth of advanced melanomas and up-regulates the activity of vascular endothelial growth factor (VEGF) and basic fibroblast growth factor (bFGF) in these cells, suggesting its role in angiogenesis [223,224].

Apart from STAT3, STAT1 has also been demonstrated to interfere melanoma development. In a mouse model for metastatic melanoma, *STAT1* knockdown impaired metastatic behavior of the cancer cells and, in melanoma cultured cells, this knockdown decreased the migration and invasive capacity of cancer cells [225]. However, another study showed that silencing *STAT1* expression is a putative mechanism by which melanoma cells evade detection by immune cells, thereby allowing cancer proliferation [226]. Overall, it is been reported that STAT1 may work as both tumor suppressor and promoter [227].

On the other hand, persistent activation of STAT5 has been associated with antitumor immunosuppression and increase in proliferation, invasion, and anti-apoptotic mechanisms of tumor cells [228,229]. Accordingly, *STAT5B* transcripts in cutaneous melanoma metastasis have been shown to be up-regulated, while 62% of melanoma metastases present increased levels of p-STAT5 when compared with normal human melanocytes [230]. It has been further suggested that a balance between *STAT1, STAT3*, and *STAT5* expression, upon interferon-mediated activation, impact the heterodimerization and transcriptional mechanisms driven by JAK-STAT activation in melanoma [231,232]. Thus, targetting STATs may represent a potential avenue for drug discovery toward melanoma treatment.

Intriguingly, the modulatory protein SOCS has been shown to exert both oncogenic and tumor suppressor roles, depending on the cellular context [233]. In melanoma, it may serve as a marker for tumor progression since *SOCS1* silencing leads to inhibition of metastasis and reversal of tumorigenic phenotype *in vitro*. In the near future, SOCS1 inhibition may also serve as an effective anti-melanoma immune effector [234,235].

### JAK-STAT pathway as a therapeutic lead for melanoma treatment

There is an increasing interest in developing new melanoma therapies based on the modulation of JAK-STAT pathway [236]. Although there is no specific drug approved aiming JAK-STAT downstream components, immunotherapies against receptors that may initiate this signaling cascade have been used as main therapy or co-adjuvant in classical chemotherapies [237].

Some of these drugs include the anti-PD-1 agents pembrolizumab and nivolumab [238]. Engagement of PD-1 by its ligands PD-L1 or PD-L2 transduces a signal that inhibits T-cell proliferation, cytokine production, and cytolytic function. In the context of cancer, ligand binding to PD-1 on T cells functionally silences the activation of tumor-associated T cells and leads to impaired cell survival and effector function, producing a tumor-permissive microenvironment [239]. Therefore, the blockage of PD-1 by monoclonal antibodies favors the recognition of the cancer cell by targetting effector T cells to induce T-cell-mediated cytotoxicity of malignant cells [239,240]. In this manner, the anti-PD1 nivolumab has shown 43% of 2-year survival rates in advanced melanoma patients [240].

Furthermore, aberrant DNA methylation in *SOCS* genes have been reported in interferon γ response in melanoma, reaching 75% in *SOCS1*, 44% in *SOCS2*, and 60% in *SOCS3* [241]. In this manner, DNA methylation and epigenetic mechanisms have been well studied to search for potential biomarkers and new therapies for melanoma [241]. There are clinical trials under recruiting status (NCT02816021) that will investigate the potential synergistic effects of hypomethylating agents, such as azacitidine, with pembrolizumab in melanoma patients [241]. Pembrolizumab has also been tested in combination with atacitinib, a JAK1 inhibitor, to treat advanced solid tumors including advanced melanoma (NCT02646748).

In human melanoma cell lines and primary cultures, curcumin, the primary bioactive component isolated from turmeric and used as dietary spice [242], induces apoptosis and modulates cellular responses to immunotherapeutic cytokines by suppressing STAT3 phosphorylation [243,244]. Further studies are necessary in order to confirm the role of curcumin as a potential new antitumorigenic and chemopreventive agent.

## TGF-β pathway

TGF-β signaling has important roles in embryogenesis and tissue homeostasis, regulating cell proliferation, migration, differentiation, and the synthesis of extracellular matrix [245–247]. Particularly in the skin, TGF-β is important for the wound healing process, especially in burn wounds [248]. TGF-β is also characterized by other cellular functions, such as cell growth, adhesion, recognition, cell fate determination, and apoptosis [249,250].

The TGF-β superfamily of ligands is evolutionarily conserved in metazoans [251]. More than 30 TGF-β members have been described in humans, and they can be divided into different families as TGF-βs, bone morphogenetic proteins (BMPs), and Nodal. These ligands are secreted as precursor molecules that require enzymatic cleavage (by furins and other proprotein convertases) to process ligand pro-domains [252]. The mature ligands are prone to form homo or heterodimers and interact with two types of serine/threonine kinase receptors to initiate the signaling. Some ligands also require the presence of co-receptors for proper downstream signaling: TGF-β2, for instance, requires TGF-β receptor type III (TβRII)[253], and Nodal signaling is facilitated by Cripto-1 [254].

In humans, distinct type I and type II TGF-β receptors have been described. Type I receptors comprise TβRI, activin receptor IB (ActRIB/ALK-4), activin receptor IA (ActRIA/ALK-2), activin receptor IC (ActRIC/ALK-7), activin receptor L1 (ActRL1/ALK-1), BMP receptor IA (BMPRIA/ALK-3) and BMP receptor IB (BMPRIB/ALK-6); while type II members are TβRII, activin receptor II (ActRII), activin receptor IIB (ActRIIB), BMP receptor II (BMPRII) and anti-Müllerian hormone receptor II (AMHRII) [255]. These receptors form heterotetrameric complexes with varied combinatins of two type I and two type II receptors and, by doing so, they signal to different transcriptional targets [247].

Smads are intracellular mediator proteins that participate on the TGF-β signaling, transducing the signal from the receptors to the nucleus. The N-terminal DNA binding domain, present in the Smad molecules, recognizes a consensus sequence containing CAGAC in the DNA, also known as Smad-binding elements (SBE) [256]. Smads can be divided in three functional classes: the first one is composed by substrates of the TGF-β receptor phosphorylation (receptor-regulated Smads, or receptor-regulated Smads (R-Smads); Smad1, 2, 3, 5, and 8); the second associates with R-Smads to transduce the signal to the nucleus (co-mediator Smad or Co-Smad; Smad4); and the third subfamily relates to the inhibitory Smads (I-Smads; Smad6 and 7), which act as antagonists of other Smads [251].

**Figure 7:**
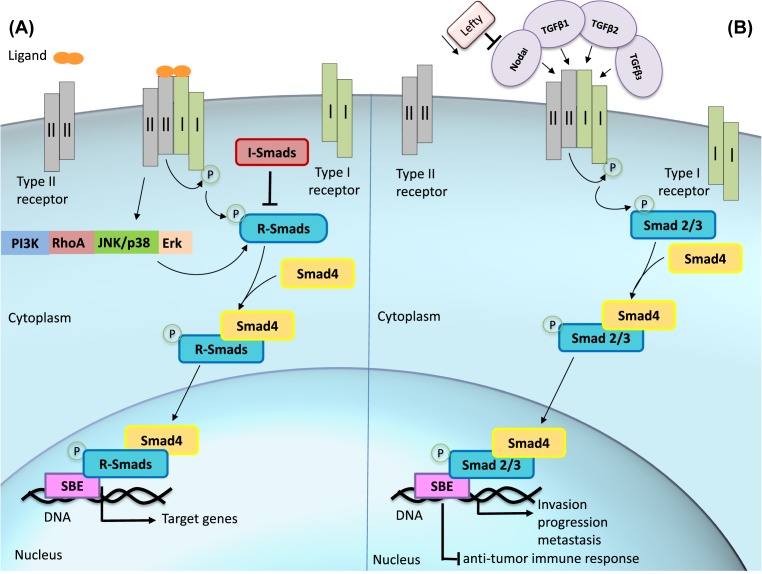
TGF-β signaling pathway in melanoma (**A**) Overview of TGF-β signaling. The ligands belonging to the TGF-β superfamily bind to type II receptor, which interacts with and phosphorylates type I receptor. R-Smads are phosphorylated by activated receptors and Co-Smad form a dimer with p-R-Smad, which then translocates into the nucleus and associates with DNA-binding cofactors to initiate transcription of target genes. (**B**) In melanoma, TGF-β ligands or Nodal can activate TGF-β signaling through Smad 2/3 to induce invasion, progression, and metastasis, and also inhibit antitumor immune response. Nodal overexpression is often related to Lefty down-regulation.

Upon ligand binding, type II receptor phosphorylates the type I receptor, exposing its Smad-binding site and enabling R-Smad activation by phosphorylation [257]. The receptor activation, driven by distinct TGF-β ligands, induces different transcription factor responses. For this, TGF-βs, Activins and Nodal activate Smad2 and 3, while BMP and growth and differentiation factors (GDFs) activate Smad1, 5, and 8 [247]. Phosphorylation of R-Smads is facilitated by a protein called Smad anchor for receptor activation (SARA), which interacts with Smad2 and Smad3 and recruits them to the receptor. The phosphorylation of Smad2 and 3 disrupts the association with SARA, enabling them to associate with Smad4 to form a dimer that is now capable of translocating into the nucleus, with the aid of importing nuclear factors [258,259]. The Smad complex activates DNA-binding nuclear cofactors such as histone acetyl transferases (HATs), p300, and CBP to initiate transcription of target genes and can eventually inhibit gene expression, depending on binding partners that may recruit histone deacetylases (HDACs) (Figure 7A) [249,256]. To keep the pathway in check, inhibitory Smad6 and 7 provide a negative feedback control of TGF-β signaling. I-Smads may act by distinct mechanisms: (i) binding to R-Smads and Smad4, and preventing their binding to the receptor or to each other; (ii) targetting the receptor for ubiquitin-dependent degradation; (iii) inducing receptor dephosphorylation; and (iv) inhibiting Smad-dependent promoter activation [249,260–264].

Activated TGF-β receptors can further stimulate other pathways in a Smad-independent manner, and these pathways can also activate Smads (like the MAPK pathway). These pathways are often altered in human tumors, in which deregulated activation increase their tumorigenic effects. For instance, TβRI can phosphorylate ShcA, promoting formation of ShcA-Grb2–Sos complex and further activation of Ras and ERK1/2. On the other hand, ERK1/2 can also phosphorylate R-Smads. TGF-β receptor can also induce Lys^63^-linked polyubiquitylation of TRAF6, activating TGF-b-activated kinase 1 (TAK1) and the IKK complex. PI3K and Akt can also be activated by TGF-β receptors and, in particular, Akt is able to regulate Smad3 activity either by sequestering it on the cytosol or by preventing its phosphorylation and degradation. Both Smad and non-Smad pathway activation seems important for epithelial to mesenchymal transition (EMT), which is associated with tumor metastasis and fibrosis [265].

### Activity of TGF-β pathway in melanoma

In the context of melanoma, TGF-β has been largely implicated in disease progression, antitumor immune response, invasiveness, and tissue remodeling [266]. Moreover, melanoma cells often metastasize to bone structures, and this ability has been also correlated to TGF-β expression [267]. Intriguingly, TGF-β signaling has opposing roles during carcinogenesis. At earlier stages, it has a tumor suppressive role by stimulating pro-apoptotic genes in response to oncogenic mutations [256,268,269]. In contrast, at more advanced stages, TGF-β signaling may act as promoter of tumor growth, since melanoma cells acquire resistance to TGF-β antiproliferative activity but inducing normal Smad signaling [270]. This effect may be due to the high expression of SKI and Sno proteins in melanoma cells, which are capable of interfering with Smad-dependent transcription of p21, responsible for cell cycle arrest [271].

The three TGF-β isoforms (TGF-β1, TGF-β2, and TGF-β3) are encoded by distinct genes and exert similar biological functions, such as growth inhibition and immunosuppression (Figure 7B) [272]. TGF-β isoforms 1, 2, and 3 have distinct expression profiles, depending on the cell type and/or tumor grade. Melanocytes normally express more TGF-β1 and do not express significant levels of TGF-β2 and TGF-β3, while advanced primary and metastatic melanomas express high levels of TGF-β2 and TGF-β3, therefore enabling a distinction between healthy and transformed cells [273]. TGF-β2 also seems to be more related to progression and tumor invasion than to initiating events [274], since patients with metastatic melanoma show increased plasma levels of both TGF-β1 and TGF-β2 [275]. In addition, TGF-β1 was capable of influencing tumor stroma (derived from human melanoma) into SCID mice, inducing fibroblasts to produce matrix in the tumor, decreasing cell death and necrosis and, consequently, promoting higher tumor growth and metastasis [276].

TGF-β is also able to repress antitumor immune response by (i) suppressing T-cell differentiation into effector T cell, (ii) enhancing regulatory T cells (Tregs) and inducing FOXP3, (iii) suppressing the generation of tumor-specific cytotoxic T lymphocytes (CTLs), and B-cell and NK-cell proliferation and function, and (iv) promoting differentiation of type II macrophages (M2), which has anti-inflammatory and tissue remodeler response [277].

Nodal is crucial for normal development, but its expression is normally ceased in adulthood [278]. Lefty, which is a feedback inhibitor of Nodal ligands, is also crucial for normal development [279]. Still, aggressive melanoma, which has capacity of invasion, migration, and vasculogenic mimicry [280], has been shown to present high expression of Nodal (Figure 7B), which is correlated with advanced tumor progression and a metastatic phenotype. Thus, Nodal has been suggested as a biomarker for melanoma progression and even a promising target for metastatic melanoma [281].

### TGF-β pathway as a therapeutic lead for melanoma treatment

Drugs that are capable of inhibiting TGF-β signaling have been considered as adjuvants for both chemotherapy and radiotherapy treatments in a broad range of cancers. Mechanistically, TGF-β inhibitors may affect the signaling cascade at different levels: (i) by targetting the receptor phosphorylation, (ii) by inhibiting the expression of TGF-β members, or (iii) by disturbing the binding of TGF-β ligands to respective receptors [282–285].

SD208 is a TGF-β receptor I kinase inhibitor that blocks the biological effects of TGF-β1 and TGF-β2 [283]. SD208 is known as a potent inhibitor of migration and invasion, even enhancing the immunogenicity of certain tumor cells (glioma) by reducing the TGF-β bioactivity [283]. *In vivo*, SD208 has prevented the development of osteolytic bone metastases originated from melanoma and, in pre-established bone lesions, the size of metastases reduced after treatment with this inhibitor [267].

GC1008 is an anti-TGF-β monoclonal antibody capable of neutralizing all human isoforms of TGF-β, and it has demonstrated a prominent anticancer activity. However, phase I clinical trials (NCT00356460) have shown side effects, including the development of keratoacanthoma lesions and squamous cell carcinomas in melanoma patients (following the blockade of TGF-β). These adverse events occurred after patients received either a complete treatment or when the maximum dose of GC1008 was administered [284,285].

Knockdown procedure (gene silencing) is another approach considered to treat TGF-β-overexpressing tumors. In particular, abnormal *TGF-β2* expression has been related to invasiveness, metastasis, and tumor progression of melanoma [274,286], thus proving to be a potential therapeutic target. Trabedersen/AP-12009 is a phosphorothioate antisense oligodeoxynucleotide that targets *TGF-β2* mRNA, which has been shown to suppress *TGF-β2* expression and then reverses the TGF-β2-mediated immunosuppressive activity of human pancreatic cancer cells. It has also reduced metastasis and angiogenesis *in vivo* [287]. Targetted treatments for patients with anaplastic astrocytoma, glioblastoma, pancreatic carcinoma, malignant melanoma, and colorectal carcinoma are currently being tested in phase I clinical trials (NCT00844064) [288].

Another approach utilized against TGF-β-overexpressing tumors relates to the autologous tumor cell vaccine called FANG™/Vigil™ (Gradalis), which incorporates a plasmid encoding granulocyte M-CSF (GM-CSF) (enhancing dendritic cell (DC) maturation and consequent tumor antigen presentation to T cells) and a novel bifunctional shRNA interference (bi-shRNAi) directed against pro-protein convertase furin, thus down-regulating TGF-β1 and β2 and reverting immunosuppressive response [282]. Vigil™ was tested as single agent for the treatment of advanced metastatic melanoma or in combination with PD-1 inhibitor Pembrolizumab in phase 2 trials (NCT01453361, NCT02574533), and has demonstrated no major side effects [273].

## Notch pathway

The Notch pathway is another highly conserved cellular pathway, present in most of the multicellular organisms, that plays an important role in cell fate determination, proliferation, differentiation, and survival [289]. The Notch signaling cascade modulates a broad range of cellular processes, including cell cycle arrest regulation, apoptosis/survival, differentiation, and stem cell maintenance, as well as the cross-talk with hypoxia response [290,291]. Consequently, aberrant Notch function has been involved in a series of human diseases, including developmental disorders, neurodegenerative diseases, and cancer (e.g. T-cell leukemia, multiple sclerosis, lymphoma, melanoma) [292–294].

**Figure 8:**
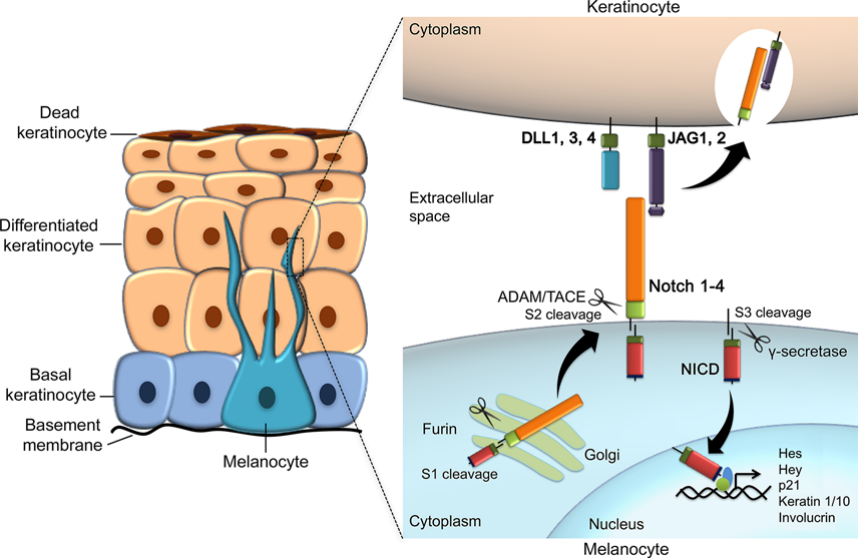
Overview of the Notch signaling pathway Initially, the Notch receptor is translocated to the trans-Golgi network as a full-length protein which is cleaved to form membrane-attached heterodimeric receptor. The signaling cascade initiates after binding of the ligands JAG1 and 2 or DSL 1, 3, and 4 to the Notch receptor between neighboring cells. This binding initiates two successive proteolytic cleavages that culminate in the release of the NICD, which is translocated into the nucleus, where it interacts with the transcription factors. In normal skin, Notch signaling is expressed in all epidermal layers and is fundamental to keratinocyte differentiation. The downstream effectors of Notch-mediated epidermal homeostasis include Hes, Hey, p21, Keratin1/10, and Involucrin Abbreviation: DSL, Delta/Serrate/Lag-2 domain.

The Notch protein family is composed by cell surface receptors that transduce signals by interacting with the transmembrane ligands Delta-like (DLL) and Jagged (JAG) on neighboring cells [295] (Figure 8). In mammals, a total of four Notch receptors (Notch1-4) and five ligands have been characterized (DLL1, 3, 4, and JAG1 and 2) [296,297]. The Notch receptors are composed by two major domains: an extracellular domain (Notch extracellular domain (NECD)) and an intracellular domain (Notch intracellular domain (NICD)), interconnected by a TM. The NICD possesses two NLS motifs at the C-terminal of the Notch TM, hich are responsible by the nuclear entry [298].

All Notch receptors are synthesized as full-length proteins that undergo successive proteolytic cleavages, culminating in the release of NICD, which will act in the nucleus. Notch ligands located in neighboring cells have a Delta/Serrate/Lag-2 (DSL) domain that mediates ligand-receptor binding and, therefore, promotes Notch signaling activation [296,299]. The triggering mechanism of the canonical Notch signaling involves proteolytic cleavages at three sites of the Notch protein: S1, S2, and S3 [300] (Figure 8). The first cleavage (S1) is mediated by furin-like convertases, which occurs in the trans-Golgi network during Notch secretion process. This cleavage generates two subunits (NECD and NICD) interconnected by the TM [7]. These processed subunits are then transported to the plasma membrane, where they associate as non-covalently bound heterodimers to form the functional Notch receptor. Following ligand binding to the receptor, the latter undergoes the second cleavage (S2) which is catalyzed by a member of A disintegrin and metalloproteases (ADAMs) family (ADAM17 or ADAM10), also known as tumor necrosis factor-α converting enzyme (TACE). S2 cleavage promotes the dissociation of the membrane-tethered intracellular Notch domain from the extracellular domain. This intracellular domain is a constitutive substrate for the final S3 cleavage [301], culminating in the release of soluble and active NICD [300], which is targetted to the nucleus by its NLS. S3 is regulated by a presenilin-dependent γ-secretase protease complex, which consists of an integral membrane protein complex [296]. After translocating to the nucleus, NICD binds to CSL (transcriptional repressor (RBPJk/CSL)), a transcriptional repressor that acts as a DNA-binding adaptor and helps to recruit the adaptor protein Mastermind-like (MAML), which in turn summon the transcriptional co-activator p300 and other components of the transcription machinery [295,301–303].

The transcriptional activation complex, composed by CSL, NICD, MAML, and p300, regulates the transcription of downstream genes such as *HES1* and *HEY* [301,304]. The Hes family of repressors act downstream of Notch signaling pathway, antagonizing the expression of a variety of transcription factors, such as Ascl1, Atoh1, and Neurog3, whose function is to maintain cells in an undifferentiated state [291]. Hes repressor genes such as *HES1* play fundamental roles in maintaining progenitor cells in an undifferentiated state [305]. The concomitant existence of transcriptional activators and repressors downstream of Notch signaling cascade play a crucial role in the diverse outcomes of this pathway.

A number of studies have also demonstrated the existence of non-canonical activation of Notch signaling in diverse cell types [306,307]. At least three types of non-canonical Notch activation have been described, which may depend or not on: (i) ligand interaction, (ii) γ-secretase activity, and (iii) the action of RBPJk/CSL complexes [308]. Both RBPJ- and Hes-independent non-canonical cascades have important functions downstream of Notch signaling, although the exact molecular events mediating these subpathways are not fully understood [291].

Notch signaling may also interact with other pathways such as PI3K, mTORC2, Wnt, NF-κβ, YY1, or HIF-1α, at cytoplasmic and/or nuclear levels [308]. Direct interactions of NICD with IKKα in the NF-κB pathway or LEF1 in the Wnt pathway have been reported, and that Notch can activate integrin via Ras, independent of binding RBPJ [304]. While many normal cellular processes (e.g. homeostatic regulation of melanocytes) require canonical Notch signaling [309], many pathological conditions including cancer and activation of the immune system are associated with Notch non-canonical signals [308]. However, these non-canonical cascades still require more detailed understanding [308].

### Activity of Notch pathway in melanoma

In healthy skin, Notch signaling is expressed in all epidermal layers and is fundamental for keratinocyte differentiation [302]. More than that, Notch signaling affects a broad range of cellular activities. These activities are impacted by the cross-talk with signaling pathways involved in different cellular events, which include cell cycle arrest, apoptosis, and survival [291]. An example of the versatile function of Notch pathway is related to epidermal development, where Notch signaling is able to stimulate the differentiation of granular cells into spinous cells and, at the same time, prevents their premature differentiation [309]. These diverse results and versatility of functions can be explained by the concomitant existence of a transcriptional activator and a repressor downstream of Notch [291]. In fact, Notch exerts roles in both proliferation and differentiation during epidermal homeostasis, since Notch pathway participates in the formation of the epidermal barrier, by promoting detachment of basal membrane cells, and activating differentiation genes and terminal keratinocyte differentiation by inducing the expression of the cell cycle-related factor p21 [302]. Alternatively, Notch signaling (via Hes1 transcription factor) maintains the survival of melanoblasts and melanocyte stem cells by preventing apoptosis initiation [310]. Both epidermal and non-epidermal melanocytes originate from melanoblasts, and it has been demonstrate that Notch signaling is essential for the maintenance of epidermal melanocytes, playing a putative role in the adaptation of melanoblasts to the epidermal environment [291,310].

Notch signaling is capable of regulating many aspects of melanomagenesis. Comparative analyses of common melanocytic nevi, dysplastic nevi, and melanomas have demonstrated an increased expression of Notch1, Notch2, and their ligands, indicating that a positive regulation of these components can be related to the melanoma progression [311]. An essential role for Notch pathway has been validated in melanoblast development as well as in melanoma progression [312]. In particular, Notch1 has been considered a primary tumorigenic factor in melanoma [313]. Melanomas re-express Notch1 and depend on the respective signaling for their growth and survival. In addition, the up-regulation of Notch1 and its target genes occurs in metastatic melanoma [314].

The interaction with other signaling cascades, such as MAPK, PI3K-Akt, NF-κB, and p53 [315], may contribute to the plural effects of Notch in melanoma. The amplification of Notch signaling may occur by increasing γ-secretase activity or by inducing the expression of Notch ligands, and potentially leads to inhibition of apoptosis [316]. Transcriptional targets of Notch signaling which are responsible for angiogenesis, proliferation, metastasis, and cell survival in tumor cells include Hes, Hey, Cyclin D1, NF-κB, STAT3, and p21.

### Notch pathway as a therapeutic lead for melanoma treatment

Considering the effects of Notch overexpression in melanoma development, the manipulation of this pathway appears to have a great therapeutic value. Several strategies to inhibit Notch pathway have been used against melanoma and other types of cancers [317]. Recent evidences have also suggested that Notch signaling is one of the most important cellular pathways in drug-resistant tumor cells [318]. In fact, down-regulation of Notch pathway may induce drug sensitivity, leading to increased inhibition of cancer cell growth, invasion, and metastasis [318]. For instance, the use of hairpin RNA targetting *NOTCH2* have shown potential to reduce tumor invasion and growth in uveal melanoma, a common intraocular malignant tumor in adults [7,319].

Two major classes of Notch inhibitors are currently in early clinical development: γ-secretase inhibitors (GSIs) and monoclonal antibodies (mAbs) against Notch receptors or their ligands [320]. The inhibition of Notch by γ-secretase inhibitor has been shown to decrease melanoma growth [316]. The GSI N-[N-(3,5-difluorophenacetyl-l-alanyl)]-S-phenylglycine t-butyl ester (DAPT) (Calbiochem) [314] is able to block the processing and further activation of all four different Notch receptors, leading to apoptotic vulnerability in melanoma cells [316]. In addition, a number of compounds targetting γ-secretase, the enzyme responsible for the S3 cleavage of Notch, are currently being tested as anticancer drugs. A recent study has shown encouraging evidence of antitumor activity in phase I clinical trial for GSI RO4929097 [321]. RO4929097 is a selective γ-secretase small molecule inhibitor with antitumor activity, tested for phase I clinical trials in combination with cediranib, a multikinase vascular endothelial growth factor receptor (VEGFR) inhibitor, in patients with solid tumors and/or melanoma [321]. The preliminary results of this study demonstrated evidence of antitumor effect with prolonged cancer stabilization in some patients [321].

Combinatorial treatments with GSIs and chemotherapy drugs for recurrent and advanced stage melanoma have been evaluated in phases I and II clinical trials. The GSI RO4929097, when co-administered with cisplatin, vinblastine, and temozolomide (NCT01196416), promotes greater elimination of tumor cells [322]. A terminated study (NCT01120275) demonstrated that RO4929097 is well tolerated by patients, and most side effects included nausea, fatigue, and anemia [323]. Although GSIs have shown great promise to treat cancer by inhibiting the Notch pathway, their prolonged use is associated with severe side effects, including gastrointestinal toxicity [320]. Another class of agents under development relates to ligand- and receptor-specific antibodies [324]. Experimental evidences have demonstrated that Notch inhibition by specific mAb against either Notch1 or Notch2 alleviates toxic effects, rather than simultaneously inhibiting both Notch receptors [320]. Notch signaling can also be interrupted due to perturbation of ligand/receptor interactions [319]. In endothelial cells, for instance, decoys leading Notch inhibition can indeed disrupt tumor growth and angiogenesis.

## Conclusion

It is clear that major signaling pathways have a seminal role in the development and maintenance of melanoma tumors. Some punctual mutations such as BRAF^V600E^ and Q61-mutant *RAS* have already been identified as favoring melanocytes to become cancer cells, therefore alterations in MAPK signaling components seem to have a primary influence on melanoma development. This pathway is also a target of new FDA approved drugs to treat metastatic melanomas. Furthermore, melanoma may trigger the over-activation of other pathways such as phosphoinositide 3-kinase (PI3K)-Akt (by reducing PTEN activity), and Wnt through non-canonical cascades, to facilitate differentiation and nutrition as well as blocking apoptotic signals in tumor cells. JAK-STAT signaling components, such as STATs, are also up-regulated in melanoma. NF-κB signaling is crucial to inflammatory responses, and inflammation has been associated with a pro-tumorigenic microenvironment, therefore promoting melanoma progression. Interestingly, the TGF-β pathway plays opposing roles in early and late stages of carcinogenesis, as tumor suppressor and tumor promoter, respectively. Nevertheless, the embryonic morphogen Nodal, a member of the TGF-β superfamily, is overexpressed in malignant melanoma and has a role in aggressiveness, being a great candidate for therapeutic target. Notch signaling plays a role in epidermal melanocyte maintenance, and aberrant expression of its components is reported not only in melanoma but in several other cancers. Experimental and clinical studies have been extensively performed to find druggable targets in each one of these pathways. It is important to reiterate that all these pathways interact to each other (via cross-talk) to create a new homeostatic status for the melanoma cells to further progress. These more complex interactions include new and yet poorly studied mechanisms to promote drug resistance and survival, which makes the drug development process more challenging. Nevertheless, as melanoma cells utilize different signaling circuitries to perpetuate the disease, increasing knowledge in this area seems equally essential to the rationale design of combined drugs, by possibly focussing on several nodes from distinct pathways, at the same time, to a more effective blockage of melanomagenesis.

## Abbreviations

APC: adenomatous polyposis coli
bFGF: basic fibroblast growth factor
BMP: bone morphogenetic protein
CBP: cAMP response element-binding protein
CDK: cyclin dependent kinase
CDKN2A: cyclin-dependent kinase inhibitor 2A
CK1α: casein kinase 1α
DVL: dishevelled
ERK: extracellular signal-related kinase
FRZD: Frizzled receptor
GAP: GTPase-activating protein
GEF: guanine nucleotide exchange factor
GPCR: G-protein-coupled receptor
Grb2: growth factor receptor-bound protein 2
GSK3β: glycogen synthase kinase 3β
GSI: γ-secretase inhibitor
IKK: inhibitor of NF-κB kinase
IL-1: interleukin-1
IκB: inhibitor of NF-κB
JAG: Jagged
JAK: Janus kinase
JNK: Jun N-terminal kinase
LEF: lymphoid enhanced transcription factor
LGR: leucine-rich repeat containing GPCR
LRP: lipoprotein receptor
MAML: mastermind-like transcriptional activator
MAP3K/MAPKKK: mitogen-activated protein kinase kinase kinase
MAPK: mitogen-activated protein kinase
MAPKK/MAP2K: MAPK kinase
MCAM: melanoma cell adhesion molecule
M-CSF: macrophage colony stimulating factor
MEK: MAPK/ERK kinase
MITF: microphthalmia-associated transcription factor
mTOR: mammalian target of rapamycin
mTORC1: mTOR protein complex 1
mTORC2: mTOR protein complex 2
NECD: Notch extracellular domain
NEMO: NF-κB essential modulator
NF1: neurofibromin 1
NF-κB: nuclear factor κ-light-chain-enhancer of activated B cell
NICD: Notch intracellular domain
NIK: NF-κB inducing kinase
NLS: nuclear localization signal
NSAID: non-steroidal anti-inflammatory drug
PD-1: programmed cell death protein 1
PD-L1 and PD-L2: programmed-death ligand 1 and 2
PH: Pleckstrin homology
PI3K: phosphoinositide 3-kinase
PIAS: protein inhibitor of activated STAT
PIP_2_: phosphatidylinositol-4,5-diphosphate
PIP_3_: phosphatidylinositol-3,4,5-trisphosphate
PTEN: phosphatase and tensin homolog
RAF: rapidly accelerated fibrosarcoma
RAS: small G-protein Ras
RBPJk/CSL: transcriptional repressor
RHD: Rel homology domain
RICTOR: rapamycin-insensitive companion of mTOR
RIP1: receptor interacting protein 1 kinase
ROR: receptor tyrosine kinase like orphan receptor
ROS: reactive oxygen species
R-Smad: receptor-regulated Smad
RTK: receptor tyrosine kinase
SARA: Smad anchor for receptor activation
SOCS: suppressor of cytokine signaling
Sos: son of Sevenless
STAT: signal transducer and activator of transcription
STC: stem cell factor
TCF: T-cell transcription factor
TCGA: The Cancer Genome Atlas
TERT: telomerase reverse transcriptase
TGF-α/β: transforming growth factor-α/β
TM: transmembrane domain
TNF-α: tumor necrosis factor α
TNFR: TNF-α receptor
TRAF: TNFR-associated factor
TSG: tumor suppressor gene
TβRII: TGF-β receptor type II
UVR: UV radiation
Vps34: vacuolar protein sorting associated 34
